# Effect of Individualized Occupational Therapy on Rehospitalization Among Patients With Schizophrenia: A Two-Year Follow-Up of a Randomized Controlled Trial

**DOI:** 10.7759/cureus.82429

**Published:** 2025-04-17

**Authors:** Takeshi Shimada, Takafumi Morimoto, Hirofumi Nagayama, Masayoshi Kobayashi

**Affiliations:** 1 Child Developmental Support Center Nanairo Karuizawa, Non-profit Organization Shiki, Nagano, JPN; 2 Scientific Department, Japanese Association of Occupational Therapists, Tokyo, JPN; 3 Department of Occupational Therapy, School of Health Sciences, Sapporo Medical University, Sapporo, JPN; 4 Department of Occupational Therapy, Kanagawa University of Human Services, Kanagawa, JPN; 5 Department of Health Sciences, Shinshu University, Nagano, JPN

**Keywords:** follow-up, occupational therapy, randomized controlled trial, rehospitalization, schizophrenia

## Abstract

Background

Schizophrenia is characterized by frequent rehospitalization. We developed an individualized occupational therapy (IOT) program to facilitate proactive participation in treatment and improve outcomes. We evaluated whether the addition of IOT to group occupational therapy (GOT) as usual care, compared to GOT alone, during hospitalization would lead to a reduction in the rehospitalization risk of schizophrenia.

Methodology

We conducted a 2-year prospective cohort study following a randomized controlled trial comparing GOT+IOT and GOT-alone groups. Participants were schizophrenia patients discharged within 1 year from psychiatric hospitals across Japan. We utilized the Brief Assessment of Cognition in Schizophrenia (BACS), Quality of Life Scale (QLS), European Quality of Life 5 Dimensions 5 Level (EQ-5D-5L), Life Assessment Scale for the Mentally Ill (LASMI), Positive and Negative Syndrome Scale (PANSS), and modified Global Assessment of Functioning for Functioning (mGAF-F) at baseline, post-treatment, and 1 and 2 years after the index discharge. We comparatively analyzed the changes in each assessment from baseline to follow-up between groups using generalized linear mixed models. We used log-rank tests to compare the distribution of time to rehospitalization and plotted the Kaplan-Meier survival estimates for time to rehospitalization by group. We estimated hazard ratios (HRs) using Cox proportional hazards models to evaluate the impact of clinical factors on rehospitalization. We used Cox regression analyses to investigate the impact of each IOT component on rehospitalization. We conducted mediation analyses to evaluate the relationship between inpatient OT type and rehospitalization and the indirect effect of medication adherence on the relationship between inpatient OT type and rehospitalization.

Results

Among 66 who met the criteria, 34 were in GOT+IOT and 32 were in GOT-alone groups. Compared to GOT alone, GOT+IOT resulted in significant improvements in changes from baseline to 1 and 2 years in BACS, QLS, EQ-5D-5L, LASMI, PANSS, and mGAF-F. Overall rehospitalization rate was 43.55%; the GOT+IOT group showed a significantly lower rate with four rehospitalized compared to 23 in the GOT-alone group. Time to rehospitalization was significantly longer for GOT+IOT than for GOT alone. The Cox proportional hazards model showed that OT type (HR=0.03), resident support persons (HR=0.32), and medication adherence (HR=0.33) were significantly associated with rehospitalization. The univariate and multivariate Cox regression analyses on the impact of IOT components on rehospitalization revealed no significant components. The inpatient OT type had a significant total effect on rehospitalization (β=0.72). The direct effect of inpatient OT type on rehospitalization was significant (β=0.64), and the indirect effect of inpatient OT type on rehospitalization via medication adherence was not significant (β=0.08).

Conclusions

Adding IOT to standard care significantly prolonged time to rehospitalization, and receiving IOT during hospitalization reduced the risk of rehospitalization for schizophrenia. Significant factors related to each IOT component that affected rehospitalization were not detected. IOT reduces the risk of rehospitalization via improved medication adherence and has a strong effect as IOT itself reduces the risk of rehospitalization. This study provides evidence that IOT could prevent rehospitalization of schizophrenia patients.

## Introduction

Schizophrenia is a chronic disease with frequent relapses and rehospitalizations during its clinical course despite continued maintenance treatment, which may lead to worse functional outcomes and long-term prognosis [1,2]. The main challenge in schizophrenia treatment is preventing relapse and rehospitalization [3], and the development of treatments is aimed at reducing these risks. To achieve this goal, individualized treatment is required for each patient. However, group treatment is considered the standard in the current medical fee system for psychiatric occupational therapy (OT) in Japan [4]. It is necessary to demonstrate the effectiveness of individually tailored OT interventions to improve the current situation of psychiatric OT in Japan and to transition from traditional OT to individualized OT intervention for improving functional outcomes and preventing rehospitalization of patients with schizophrenia [5-7].

We developed an individualized occupational therapy (IOT) program to facilitate proactive participation in treatment and improve outcomes of patients with schizophrenia [5,6]. To our knowledge, no similar IOT programs are available. It is a comprehensive intervention involving multiple psychosocial treatment components. The IOT strategy is to facilitate proactive participation in treatment, enhance cognition, and prompt adaptive behaviors to maximize functional outcomes.

A preliminary IOT study evaluated the impact of adding IOT to group occupational therapy (GOT) as standard care for rehospitalization of patients with schizophrenia in Nagano Prefecture, Japan [7]. The results of this study showed that compared to GOT alone, the addition of IOT to GOT may reduce the risk of rehospitalization of patients with schizophrenia [7]. However, this result cannot be generalized throughout Japan because it was conducted at hospitals in a limited area of Nagano Prefecture, Japan [7]. Information on the association between rehospitalization and IOT components was not available.

To address these issues, we conducted a randomized controlled trial at 14 sites across Japan to evaluate the effect of adding IOT to GOT during hospitalization compared with GOT alone [8]. We addressed the issue of which IOT component was the most strongly involved in the cognitive outcome by evaluating the IOT dosage for each IOT subprogram. The findings of that study showed that the addition of IOT to GOT led to several significantly improved cognitive domains and other outcomes compared to GOT alone in the inpatient treatment phase [8]. However, the long-term effects of IOT on the clinical outcomes and the rehospitalization risk among patients with schizophrenia have not yet been comprehensively evaluated.

The question is whether, compared to GOT alone, the addition of IOT to GOT during hospitalization would lead to the reduction of rehospitalization risk among patients with schizophrenia. To test this hypothesis, we conducted a two-year follow-up study following the same patient sample in our prior IOT study [8].

## Materials and methods

Study design and procedures

Between December 2020 and September 2024, a multisite two-year prospective cohort study following our prior study [8] was conducted at Medical Corporation Seitaikai, Mental Support Soyokaze Hospital, Nagano, Japan; Specified Medical Corporation, Hayashishita Hospital, Sapporo, Japan; Kinan Mental Medical Center, Wakayama, Japan; National Hospital Organization Sakakibara Hospital, Mie, Japan; Medical Corporation Okakai, Clinic Day-care Center, Kyoto, Japan; Medical Corporation Yuaikai, Tikumaso Mental Hospital, Nagano, Japan; Iwamizawa Municipal General Hospital, Iwamizawa, Japan; Kobe University Hospital, Hyogo, Japan; Nagoya University Hospital, Nagoya, Japan; Medical Corporation Kawasakikai, Mizuma Hospital, Osaka, Japan; Social Medical Corporation Mikamikai, Higashikouri Hospital, Osaka, Japan; Social Medical Corporation Mikamikai, Higashikouri Second Hospital, Osaka, Japan; Specified Medical Corporation Kyowakai, Kyowa Hospital, Aichi, Japan; and Okayama Psychiatric Medical Center, Okayama, Japan.

Following baseline assessments, the eligible participants were randomized to the GOT + IOT and GOT-alone groups. Our prior study [8] has described the randomization procedure. Treatment in each group lasted approximately three months from hospitalization to discharge. Assessments were conducted at baseline before randomization, at discharge or three months following hospitalization (if the hospitalization period was over 3 months), and over one and two years after the index discharge.

This study was approved by the ethics committee of the Japanese Association of Occupational Therapists (approval number: 2020001) and each trial site. All participants provided written informed consent. The study was registered with the University Hospital Medical Information Network Clinical Trials Registry (UMIN-CTR) (registration number: UMIN000042532).

Participants

Participants were recruited from all new patients hospitalized at each trial site between January 2021 and June 2022. The inclusion criteria were age 20-60 years, diagnosed with schizophrenia based on the Structured Clinical Interview for Diagnostic and Statistical Manual of Mental Disorders, Fifth Edition (DSM-5) Disorders Research Version (SCID-5-RV) [9], newly hospitalized in a psychiatric hospital, and discharged within one year. The reason we included patients who were discharged within one year was that factors prolonging hospitalization for more than one year have been reported to include not only the patients’ own medical conditions but also poor family acceptance and lack of residence [10]. The exclusion criteria were current primary DSM-5 diagnosis other than schizophrenia, a history of intellectual disability or neurological disorder, a diagnosis of substance use disorder within six months before consent, a history of psychosis accounted for by substance abuse, current risk of suicide, and the presence of a serious comorbid physical disorder, limiting the ability of the participants to participate in the intervention program and complete the assessments.

OT interventions

The OT intervention methods were reported in our prior studies [5,6]; here, we only describe the primary features. The members of the collaborative trial sites received training in IOT before starting the study and implemented the IOT intervention, based on the original IOT manual. All participants received treatment as usual over the course of the study, including a broad array of clinical interventions used to treat schizophrenia. IOT is a goal-oriented, individualized treatment provided on a one-on-one basis by occupational therapists tailored to each patient [5,6]. IOT consists of a combination of psychosocial treatment components relevant to OT practice, including motivational interviews, self-monitoring, individualized visits, craft activities, individualized psychoeducation, and discharge planning [5,6]. The main component of the program, specific to the OT profession, was the incorporation of craft activities with individualized coaching by occupational therapists, designed to address and improve cognition [5,6]. GOT is a standard activity-oriented group treatment already being implemented at each trial site and includes the following programs: physical fitness, craft activities, cooking, music, recreation, and psychoeducation [5,6]. Patients voluntarily selected any desired program from among these options and participated at an individualized rate [5,6]. Craft activities were also included in the GOT program; however, each patient voluntarily completed the craft activities based on their preferences, and occupational therapists assisted them only on request [5,6].

Information on OT interventions showed that patients in the GOT + IOT group completed an average of 61.79 (standard deviation (SD) = 38.44) OT sessions and 2,847.50 (SD = 1,479.21) OT implementation time over an average of 58.09 (SD = 26.36) days, and patients in the GOT alone group completed an average of 54.91 (SD = 12.12) OT sessions and 2,530.59 (SD = 1,307.93) OT implementation time over an average of 63.03 (SD = 28.93) days, with no significant between-group differences [8].

Outcome measures

The primary outcomes were rehospitalization over time and time to rehospitalization, which was determined as the time from index psychiatric hospital discharge to rehospitalization in a psychiatric hospital during the two-year follow-up and censored at psychiatric rehospitalization or on day 730 after index psychiatric discharge, whichever came first [7].

The following demographic data were obtained at index discharge or during the two-year follow-up directly from participants, interviews with support persons, and medical records: age, sex, onset age, total number of hospital stays, total length of hospital stays, education, marital status, comorbidity (physical and psychiatric comorbidities), length of recent hospitalization, length of OT from hospitalization, length of inpatient OT, number of inpatient OT sessions, type of inpatient OT (GOT + IOT or GOT alone), number and time spent in each IOT subprogram, resident support persons, health and welfare services, and antipsychotic medication.

The following assessment measures at baseline, post-treatment, and one and two years after the index psychiatric hospital discharge were collected from medical records: cognition assessed using the Brief Assessment of Cognition in Schizophrenia (BACS) [11]; intrinsic motivation assessed by the sum of the following three items from the Quality of Life Scale (QLS): sense of purpose, motivation, and curiosity [12]; health-related quality of life assessed using the European Quality of Life 5 Dimensions 5 Level Version (EQ-5D-5L) [13]; social functioning assessed using the Life Assessment Scale for the Mentally Ill (LASMI) [14]; psychopathology assessed using the Positive and Negative Syndrome Scale (PANSS) [15]; and functional level assessed using the modified Global Assessment of Functioning for functioning (mGAF-F) [16,17]. Table 1 provides the details of the assessment measures. The assessors were masked to treatment allocation. These assessors were trained and certified in the use of the outcome measures, and masking was successfully maintained throughout the study.

**Table 1:** Description of assessment measures.

Domain	Measurement	Description
Cognition	Brief Assessment of Cognition in Schizophrenia (BACS) [11]	The BACS comprises the following six domains: verbal memory (list learning), working memory (digit sequencing task), motor speed (token motor task), verbal fluency (category instances, letter fluency), attention and processing speed (symbol coding), and executive function (Tower of London test). Each of the six domains is standardized by creating z-scores, whereby the mean of healthy controls is set to 0, and the standard deviation (SD) is set to 1. The BACS composite z-scores are calculated as the average z-scores of each of the six BACS subdomains, which are re-normed based on the SD of the normative sample data of the same age range and sex
Intrinsic motivation	Quality of Life Scale (QLS) [12]	The sum of the following three items from the QLS: sense of purpose, motivation, and curiosity, was used. Each item was rated on a scale ranging from 0 to 6, with higher scores indicating better function
Health-related quality of life	European Quality of Life 5 Dimensions 5 Level Version (EQ-5D-5L) [13]	The EQ-5D-5L is a standardized, patient-reported, generic instrument for measuring health outcomes and provides a simple descriptive profile and a single index value for the health status. The instrument consists of the EQ-5D-5L descriptive system and the visual analog scale (VAS). The descriptive system consists of five dimensions (mobility, self-care, usual activities, pain/discomfort, anxiety/depression) with five levels of severity (no problems, slight problems, moderate problems, severe problems, extreme problems) for each dimension. These are used to generate the index score, ranging from 0 (really bad/death) to 1 (perfect health). The VAS records the respondent’s self-rated health on a 20-cm, 100-point vertical VAS with endpoints labeled “The worst health you can imagine” at 0 and “The best health you can imagine” at 100. Therefore, the VAS scores range from 0 (worst health you can imagine) to 100 (best health you can imagine)
Social functioning	Life Assessment Scale for the Mentally Ill (LASMI) [14]	The LASMI comprises 40 items in the following five categories: daily living, interpersonal relations, work, endurance and stability, and self-recognition. Each item is rated from 0 to 4, with higher scores indicating a more severe disability
Psychopathology	Positive and Negative Syndrome Scale (PANSS) [15]	The PANSS is a 30-item rating scale designed to assess the severity of psychotic symptoms and consists of the following three domains: positive, negative, and general psychopathology. Each item is rated from 1 to 7, with higher scores indicating more severe symptoms
Functional level	Modified Global Assessment of Functioning (mGAF-original) for subscale of social functioning (mGAF-F) [16,17]	Functional level is assessed with the modified Global Assessment of Functioning (mGAF-original) scale [16]. Eguchi et al. [17] translated the mGAF-original scale [16] to Japanese and developed the psychological symptom (mGAF-S) and social functioning (mGAF-F) subscales by dividing the items and anchor points of the mGAF-original scale. The mGAF-F, a single-item rating scale for measuring patient functioning, is used in this study. Scores on each scale are rated between 21 and 90, with higher scores indicating better functioning

Outpatient treatment, including outpatient OT, daycare treatment, home-visit nursing, and medication adherence, was investigated through a follow-up performed two years after the index discharge. Medication adherence was assessed by the psychiatrists in charge of each participant, who checked the adherence data and ensured the absence or presence of medication interruptions or self-adjustments based on information from the participants, their family members, or the clinicians, and was determined as good or poor.

Statistical analysis

We estimated the sample size with a power of 80% and a two-tailed α type I error rate of 5% for the hazard ratio (HR) of rehospitalization risk of 0.543 [6]. The calculation results indicated that the required sample size was 92 patients (46 patients per group). An estimated dropout rate of 5.15% was estimated [5]. A planned sample size of 96 patients (48 per group) was obtained.

To evaluate the outcomes, we used generalized linear mixed models (GLMMs) with an intention-to-treat analysis, including all available data for each outcome measure. Changes over time from baseline to follow-up in the BACS, QLS, EQ-5D-5L, LASMI, PANSS, and mGAF-F scores between the groups were analyzed using GLMMs. Participants, site, and site-by-group interaction were included as random effects, whereas age, sex, number of hospital stays, baseline scores, group (GOT + IOT or GOT alone), assessment time, and time-by-group interaction were included as fixed effects. If the baseline variables differed significantly between the groups, these were controlled as covariates. Effect sizes for intervention-related changes in each outcome measure were calculated using Cohen’s d. Bonferroni’s correction was applied to account for multiple comparisons.

We used log-rank tests to compare the distribution of time to rehospitalization in the groups and plotted the Kaplan-Meier survival estimates for the time to rehospitalization by group. We estimated HRs using Cox proportional hazards models to evaluate the impact of demographic and clinical factors on rehospitalization, after checking for proportional hazard assumptions. Based on prior studies on rehospitalization for schizophrenia, we selected the following potential factors for therapeutic intervention other than the type of inpatient OT (GOT + IOT vs. GOT alone): physical comorbidities [18], switching antipsychotics during hospitalization [19], cognition (BACS composite score at posttreatment) [7], resident support persons [7,20], and medication adherence [7,21], and these factors were entered into a multivariate Cox model, after simultaneously controlling for potential confounders. Adjustment factors for multivariate analysis were age, sex, and the number of hospital stays, which were factors used to stratify random allocation.

We also used Cox regression analyses to investigate the impact of each IOT component on rehospitalization among participants in the GOT + IOT group if the type of inpatient OT was detected as a significant factor of rehospitalization.

The significance level was set at p-values <0.05 for a two-sided test. Statistical analyses were performed using SPSS Statistics, version 28.0 (IBM Corp., Armonk, NY, USA).

Additional analysis

Using the Cox proportional hazards model, we confirmed that inpatient OT type and medication adherence were significantly associated with rehospitalization. The question is whether the addition of IOT to GOT directly or indirectly mediates improvements in medication adherence to reduce the risk of rehospitalization. To test this hypothesis, we evaluated the relationship between the type of inpatient OT and rehospitalization (Model 1) and the indirect effect of medication adherence on the relationship between the type of inpatient OT and rehospitalization (Model 2) using causal mediation analyses. Causal mediation analyses were considered valid when the significance level was maintained with 5,000 bootstrapping samples, and the 95% confidence intervals (CIs) of the indirect effect did not include zero. Differences were considered statistically significant at p-values <0.05 for two-tailed tests.

## Results

Participants

**Figure 1:**
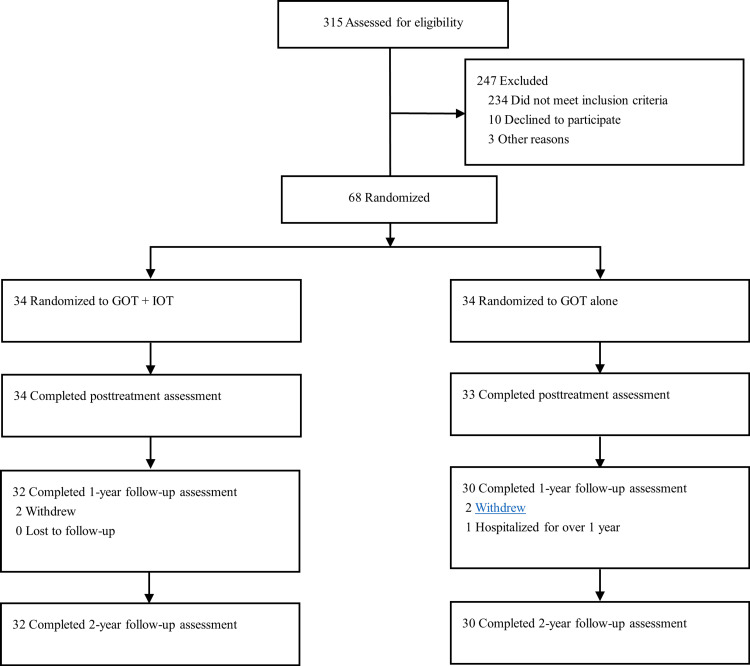
Study flowchart. GOT: group occupational therapy; IOT: individualized occupational therapy

Figure 1 shows the study flowchart, including that of our prior study [8]. Of the 315 who were assessed for eligibility, 68 who met the criteria for the prior study were randomized to the GOT + IOT (n = 34) and GOT-alone group (n = 34). Overall, 67 (98.53%) completed the trial inpatient OT intervention [8]. Of those who completed the intervention, one did not meet the criteria for this study due to hospitalization for >1 year. Of the 66 patients who met the criteria for this study (34 in GOT + IOT and 32 in GOT alone), four (two in GOT + IOT and two in GOT alone) were excluded during the follow-up period because they withdrew participation. No significant differences were found in a comparison of the participants who dropped out of the study and those who remained based on demographics and clinical characteristics. Therefore, 62 participants completed the two-year follow-up period and comprised the final sample used for the analysis. Of these, 32 (51.61%) were from the GOT + IOT group, and 30 (48.39%) were from the GOT-alone group.

Participants’ characteristics

Among the eligible participants in this study, 62 completed the two-year follow-up. Demographic data of participants showed that 39 (62.90%) had access to resident support, and 16 (25.81%) used health and welfare services (Table 2). Outpatient treatment data showed that 18 (29.03%) patients participated in outpatient OT, and 34 (54.84%) had good medication adherence (Table 3). The participants who received GOT + IOT showed better trends for each assessment measure than those who received GOT alone (Table 4).

**Table 2:** Demographic data of participants. ^a^: Collected at baseline data; ^b^: collected over a two-year follow-up; ^c^: male vs. female; ^d^: single vs. married vs. separated or divorced vs. widowed. FGA: first-generation antipsychotics; GOT: group occupational therapy; IOT: individualized occupational therapy; OT: occupational therapy; SD: standard deviation; SGA: second-generation antipsychotics

	Total		GOT + IOT		GOT alone	
Age (years), mean (SD) ^a^	44.22	(9.34)	44.97	(9.17)	43.47	(9.59)
Sex, n (% female) ^a, c^	35	(51.47)	17	(50.00)	18	(52.94)
Onset age (years), mean (SD) ^a^	23.84	(6.28)	23.47	(6.82)	24.21	(5.76)
Number of hospital stays (times), mean (SD) ^a^	5.09	(3.75)	5.26	(3.60)	4.91	(3.93)
Total length of hospital stays (months), mean (SD) ^a^	18.56	(28.01)	15.22	(11.23)	21.91	(38.00)
Education (year), mean (SD) ^a^	12.61	(2.04)	12.38	(1.52)	12.85	(2.46)
Marital status, n (% single) ^a, d^	57	(83.82)	28	(82.35)	29	(85.29)
Comorbidity, n (% existence) ^a^	16	(23.53)	6	(17.65)	10	(29.41)
Physical comorbidities, n (% existence) ^a^	15	(22.06)	5	(14.71)	10	(29.41)
Psychiatric comorbidities, n (% existence) ^a^	1	(1.47)	1	(2.94)	0	(0)
Length of recent hospitalization (day), mean (SD)	99.34	(86.53)	85.58	(58.87)	113.10	(106.61)
Living conditions after discharge ^b^						
Income, n (% yes)	49	(79.03)	25	(78.13)	24	(80.00)
Resident support persons, n (% yes)	39	(62.90)	20	(62.50)	19	(63.33)
Health and welfare services ^b^						
Nursing care benefits, n (% yes)	2	(3.23)	0	(0)	2	(6.67)
Training services, n (% yes)	8	(12.90)	4	(12.50)	4	(13.33)
Community life support, n (% yes)	1	(1.61)	1	(3.13)	0	(0)
Consultation support, n (% yes)	5	(8.06)	3	(9.38)	2	(6.67)
Antipsychotic medication						
Chlorpromazine equivalent dose, mean (SD)						
Baseline	878.29	(411.03)	841.35	(391.17)	915.24	(432.64)
Posttreatment	796.04	(367.84)	776.71	(338.80)	815.38	(398.95)
One-year follow-up	745.62	(239.01)	713.18	(183.24)	778.06	(283.25)
Two-year follow-up	732.06	(237.35)	693.76	(187.65)	770.35	(275.94)
Generation, n (%) ^a^						
FGA	0	(0)	0	(0)	0	(0)
SGA	63	(92.65)	31	(91.18)	32	(94.12)
FGA + SGA	5	(7.35)	3	(8.82)	2	(5.88)
Prescription, n (%) ^c^						
Monotherapy	18	(26.47)	8	(23.53)	10	(29.41)
Polypharmacotherapy	50	(73.53)	26	(76.47)	24	(70.59)
Switching antipsychotics during hospitalization, n (% no)	48	(70.59)	22	(64.71)	26	(76.47)

**Table 3:** Outpatient treatment data of participants. ^a^: Collected over a two-year follow-up; ^b^: good vs. poor. GOT: group occupational therapy; IOT: individualized occupational therapy; OT: occupational therapy; SD: standard deviation

	Total		GOT + IOT		GOT alone	
Outpatient OT, n (% yes)^ a^	18	(29.03)	14	(43.75)	4	(13.33)
Day-care treatment, n (% yes) ^a^	17	(27.42)	9	(28.13)	8	(26.67)
Home-visit nursing, n (% yes) ^a^	18	(29.03)	9	(28.13)	9	(30.00)
Medication adherence, n (% good) ^a, b^	34	(54.84)	21	(65.63)	13	(43.33)

**Table 4:** Assessment data at baseline, posttreatment, one-year follow-up, and two-year follow-up. BACS: Brief Assessment of Cognition in Schizophrenia; GOT: group occupational therapy; IOT: individualized occupational therapy; LASMI: Life Assessment Scale for the Mentally Ill; mGAF-F: modified Global Assessment of Functioning social functioning subscale; PANSS: Positive and Negative Syndrome Scale; QLS: Quality of Life Scale; SD: standard deviation; VAS: Visual Analog Scale

	Total		GOT + IOT		GOT alone	
	Mean	(SD)	Mean	(SD)	Mean	(SD)
BACS verbal memory						
Baseline	-1.63	(0.98)	-1.72	(1.00)	-1.53	(0.97)
Posttreatment	-0.71	(0.78)	-0.56	(0.63)	-0.88	(0.88)
One-year follow-up	-0.95	(1.10)	-0.61	(0.70)	-1.30	(1.31)
Two-year follow-up	-0.89	(1.16)	-0.75	(0.93)	-1.03	(1.34)
BACS working memory						
Baseline	-1.31	(1.08)	-1.46	(1.08)	-1.17	(1.07)
Posttreatment	-0.53	(0.13)	-0.21	(1.00)	-0.86	(1.00)
One-year follow-up	-0.64	(1.30)	-0.27	(1.36)	-1.01	(1.14)
Two-year follow-up	-0.65	(1.29)	-0.31	(1.42)	-1.01	(1.03)
BACS motor speed						
Baseline	-3.02	(1.69)	-2.60	(1.65)	-3.44	(1.65)
Posttreatment	-2.12	(1.48)	-1.49	(1.11)	-2.78	(1.55)
One-year follow-up	-2.46	(1.46)	-1.91	(1.33)	-3.03	(1.39)
Two-year follow-up	-2.55	(1.78)	-2.07	(1.71)	-3.05	(1.73)
BACS verbal fluency						
Baseline	-1.10	(1.17)	-1.22	(0.95)	-0.98	(1.36)
Posttreatment	-0.45	(1.15)	-0.22	(1.01)	-0.69	(1.26)
One-year follow-up	-0.56	(1.27)	-0.10	(1.08)	-1.04	(1.29)
Two-year follow-up	-0.57	(1.34)	-0.18	(1.35)	-0.97	(1.23)
BACS attention						
Baseline	-2.36	(1.17)	-2.33	(1.04)	-2.38	(1.31)
Posttreatment	-1.25	(0.90)	-0.73	(0.69)	-1.78	(0.77)
One-year follow-up	-1.70	(1.28)	-1.05	(1.00)	-2.37	(1.20)
Two-year follow-up	-1.56	(1.37)	-0.87	(1.05)	-2.28	(1.30)
BACS executive function						
Baseline	-1.65	(1.77)	-1.92	(1.32)	-1.39	(2.12)
Posttreatment	-0.45	(1.16)	-0.18	(0.86)	-0.72	(1.37)
One-year follow-up	-0.77	(1.94)	-0.28	(1.64)	-1.27	(2.12)
Two-year follow-up	-0.58	(1.56)	-0.18	(0.85)	-1.00	(1.98)
BACS composite score						
Baseline	-3.40	(1.92)	-3.34	(1.40)	-3.46	(2.35)
Posttreatment	-1.64	(1.45)	-0.98	(1.09)	-2.30	(1.48)
One-year follow-up	-2.17	(2.51)	-0.76	(1.24)	-1.66	(1.09)
Two-year follow-up	-1.98	(2.18)	-1.19	(1.62)	-2.77	(2.40)
QLS						
Baseline	7.78	(3.31)	6.76	(2.28)	8.79	(3.85)
Posttreatment	10.79	(3.14)	11.47	(2.27)	10.09	(3.75)
One-year follow-up	10.03	(3.07)	11.31	(2.39)	8.75	(3.16)
Two-year follow-up	10.91	(3.52)	12.18	(2.71)	9.55	(3.80)
EQ-5D-5L Index score						
Baseline	0.73	(0.19)	0.72	(0.22)	0.74	(0.17)
Posttreatment	0.84	(0.16)	0.87	(0.14)	0.80	(0.17)
One-year follow-up	0.84	(0.13)	0.88	(0.10)	0.81	(0.15)
Two-year follow-up	0.86	(0.15)	0.91	(0.10)	0.81	(0.18)
EQ-5D-5L VAS score						
Baseline	56.75	(25.04)	56.47	(26.53)	57.03	(23.85)
Posttreatment	71.15	(20.05)	74.03	(20.01)	68.18	(19.97)
One-year follow-up	68.59	(16.12)	74.69	(11.14)	62.50	(18.10)
Two-year follow-up	72.27	(19.23)	80.45	(12.59)	63.55	(21.34)
LASMI daily living						
Baseline	1.71	(1.06)	1.72	(1.07)	1.70	(1.06)
Posttreatment	1.29	(0.92)	1.01	(0.68)	1.58	(1.05)
One-year follow-up	1.17	(0.96)	0.98	(0.85)	1.36	(1.03)
Two-year follow-up	1.15	(0.91)	0.99	(0.85)	1.31	(0.96)
LASMI interpersonal relations						
Baseline	1.57	(0.95)	1.63	(0.91)	1.52	(1.01)
Posttreatment	1.20	(0.75)	0.94	(0.51)	1.47	(0.86)
One-year follow-up	1.18	(0.90)	0.97	(0.67)	1.40	(1.06)
Two-year follow-up	1.27	(0.89)	1.14	(0.69)	1.41	(1.05)
LASMI work						
Baseline	2.14	(0.79)	2.42	(0.59)	1.86	(0.89)
Posttreatment	1.57	(1.36)	1.37	(1.64)	1.78	(0.96)
One-year follow-up	1.47	(0.92)	1.25	(0.80)	1.69	(1.00)
Two-year follow-up	1.54	(0.91)	1.36	(0.85)	1.74	(0.95)
LASMI endurance and stability						
Baseline	3.72	(1.15)	3.93	(1.31)	3.51	(0.94)
Posttreatment	3.78	(1.68)	4.10	(1.81)	3.45	(1.48)
One-year follow-up	3.56	(1.57)	3.49	(1.09)	3.63	(1.96)
Two-year follow-up	3.51	(1.43)	3.66	(1.39)	3.35	(1.47)
LASMI self-recognition						
Baseline	2.31	(1.06)	2.40	(1.08)	2.22	(1.04)
Posttreatment	1.83	(1.04)	1.74	(0.99)	1.93	(1.09)
One-year follow-up	1.58	(1.00)	1.40	(0.76)	1.77	(1.18)
Two-year follow-up	1.75	(1.03)	1.71	(0.85)	1.79	(1.21)
PANSS positive						
Baseline	22.63	(8.08)	23.03	(7.63)	22.24	(8.60)
Posttreatment	18.37	(8.39)	17.53	(6.64)	19.24	(9.91)
One-year follow-up	18.16	(7.79)	17.12	(6.10)	19.26	(9.24)
Two-year follow-up	19.70	(7.88)	18.85	(6.37)	20.61	(9.25)
PANSS negative						
Baseline	24.50	(6.78)	25.62	(5.32)	23.38	(7.91)
Posttreatment	17.12	(7.34)	14.03	(3.92)	20.30	(8.63)
One-year follow-up	17.87	(7.73)	15.30	(5.67)	20.61	(8.74)
Two-year follow-up	19.92	(7.90)	17.61	(5.78)	22.39	(9.11)
PANSS general psychopathology						
Baseline	46.26	(15.15)	45.97	(15.04)	46.56	(15.48)
Posttreatment	39.31	(15.68)	38.56	(14.77)	40.09	(16.76)
One-year follow-up	39.19	(16.69)	36.94	(13.99)	41.58	(19.10)
Two-year follow-up	41.11	(15.36)	40.45	(14.38)	41.81	(16.54)
PANSS total						
Baseline	93.69	(27.92)	94.76	(25.44)	92.62	(30.54)
Posttreatment	73.71	(30.54)	70.12	(23.96)	77.29	(35.95)
One-year follow-up	75.20	(30.18)	69.27	(23.47)	81.52	(35.29)
Two-year follow-up	80.73	(29.36)	76.91	(24.68)	84.81	(33.58)
mGAF-F						
Baseline	41.34	(10.11)	42.06	(9.58)	40.62	(10.70)
Posttreatment	45.54	(13.39)	50.35	(7.45)	40.58	(16.21)
One-year follow-up	50.59	(12.20)	54.97	(7.86)	45.94	(14.25)
Two-year follow-up	51.36	(12.58)	54.88	(7.82)	47.61	(15.46)

Results of outcome measures

There were no significant differences in the baseline assessment results between groups (Table 5). Additionally, no significant site effects or site-by-group interactions were observed in any outcome score. Table 5 presents the between-group differences in the changes from the baseline to follow-up assessments.

**Table 5:** Changes in outcome measures from baseline. ^*^: p < 0.05; ^**^: p < 0.01. ^a^: the p-value is the test for the t-statistic of comparing groups; ^b^: the p-value is the test for the F-statistic of interaction of time and treatment group. BACS: Brief Assessment of Cognition in Schizophrenia; GOT: group occupational therapy; IOT: individualized occupational therapy; LASMI: Life Assessment Scale for the Mentally Ill; mGAF-F, modified Global Assessment of Functioning social functioning subscale; NA: not applicable; PANSS: Positive and Negative Syndrome Scale; QLS: Quality of Life Scale; SD: standard deviation; VAS: Visual Analog Scale

	GOT + IOT		GOT alone							
	Mean	(SD)	Mean	(SD)	P-value ^a^	Estimate	(95% CI)	P-value ^b^	Cohen’s d	(95% CI)
BACS verbal memory										
Baseline	-1.72	(1.00)	-1.53	(0.97)	0.415	NA		NA	NA	
Posttreatment	-0.56	(0.63)	-0.88	(0.88)	0.087	-1.17	(-1.97 to -0.38)	0.021	0.42	(-0.06 to 0.91)
One-year follow-up	-0.61	(0.70)	-1.30	(1.31)	0.011	-1.07	(-2.21 to 0.07)	0.001	0.65	(0.15 to 1.15)
Two-year follow-up	-0.75	(0.93)	-1.03	(1.34)	0.338	-1.01	(-2.06 to 0.04)	0.004	0.24	(-0.25 to 0.73)
BACS working memory										
Baseline	-1.46	(1.08)	-1.17	(1.07)	0.275	NA		NA	NA	
Posttreatment	-0.21	(1.00)	-0.86	(1.00)	0.010	-0.93	(-1.68 to -0.17)	<0.001	0.65	(0.16 to 1.14)
One-year follow-up	-0.27	(1.36)	-1.01	(1.14)	0.023	-0.83	(-1.78 to 0.12)	<0.001	0.58	(0.08 to 1.08)
Two-year follow-up	-0.31	(1.42)	-1.01	(1.03)	0.029	-0.79	(-1.78 to 0.20)	<0.001	0.56	(0.06 to 1.06)
BACS motor speed										
Baseline	-2.60	(1.65)	-3.44	(1.65)	0.050	NA		NA	NA	
Posttreatment	-1.49	(1.11)	-2.78	(1.55)	<0.001	-2.57	(-3.62 to -1.53)	0.119	0.96	(0.45 to 1.47)
One-year follow-up	-1.91	(1.33)	-3.03	(1.39)	0.002	-2.54	(-3.67 to -1.41)	0.270		(0.31 to 1.34)
Two-year follow-up	-2.07	(1.71)	-3.05	(1.73)	0.025	-2.55	(-3.87 to -1.22)	0.487	0.82	(0.07 to 1.07)
BACS verbal fluency										
Baseline	-1.22	(0.95)	-0.98	(1.36)	0.414	NA		NA	NA	
Posttreatment	-0.22	(1.01)	-0.69	(1.26)	0.094	-0.80	(-1.61 to 0.01)	0.002	0.42	(-0.07 to 0.90)
One-year follow-up	-0.10	(1.08)	-1.04	(1.29)	0.003	-0.73	(-1.68 to 0.22)	<0.001	0.79	(0.28 to 1.30)
Two-year follow-up	-0.18	(1.35)	-0.97	(1.23)	0.018	-0.70	(-1.76 to 0.37)	<0.001	0.61	(0.11 to 1.12)
BACS attention										
Baseline	-2.33	(1.04)	-2.38	(1.31)	0.858	NA		NA	NA	
Posttreatment	-0.73	(0.69)	-1.78	(0.77)	<0.001	-1.81	(-2.63 to -0.98)	<0.001	1.44	(0.90 to 1.98)
One-year follow-up	-1.05	(1.00)	-2.37	(1.20)	<0.001	-1.77	(-2.70 to -0.84)	<0.001	1.20	(0.66 to 1.74)
Two-year follow-up	-0.87	(1.05)	-2.28	(1.30)	<0.001	-1.71	(-2.68 to -0.74)	<0.001	1.20	(0.66 to 1.73)
BACS executive function										
Baseline	-1.92	(1.32)	-1.39	(2.12)	0.221	NA		NA	NA	
Posttreatment	-0.18	(0.86)	-0.72	(1.37)	0.059	-1.05	(-2.27 to 0.16)	0.001	0.47	(-0.02 to 0.95)
One-year follow-up	-0.28	(1.64)	-1.27	(2.12)	0.042	-0.92	(-2.43 to 0.60)	<0.001	0.52	(0.02 to 1.02)
Two-year follow-up	-0.18	(0.85)	-1.00	(1.98)	0.036	-0.81	(-2.28 to 0.67)	<0.001	0.54	(0.04 to 1.04)
BACS composite score										
Baseline	-3.34	(1.40)	-3.46	(2.35)	0.798	NA		NA	NA	
Posttreatment	-0.98	(1.09)	-2.30	(1.48)	<0.001	-1.40	(-2.10 to -0.71)	<0.001	1.01	(0.50 to 1.51)
One-year follow-up	-0.76	(1.24)	-1.66	(1.09)	0.010	-2.39	(-4.08 to -0.71)	<0.001	0.76	(0.23 to 1.29)
Two-year follow-up	-1.19	(1.62)	-2.77	(2.40)	0.002	-2.27	(-3.95 to -0.59)	<0.001	0.78	(0.27 to 1.27)
QLS										
Baseline	6.76	(2.28)	8.79	(3.85)	0.061	NA		NA	NA	
Posttreatment	11.47	(2.27)	10.09	(3.75)	0.072	9.42	(7.32 to 11.52)	<0.001	0.45	(-0.04 to 0.93)
One-year follow-up	11.31	(2.39)	8.75	(3.16)	<0.001	9.78	(7.20 to 12.37)	<0.001	0.91	(0.40 to 1.43)
Two-year follow-up	12.18	(2.71)	9.55	(3.80)	0.002	10.16	(7.44 to 12.88)	<0.001	0.80	(0.29 to 1.31)
EQ-5D-5L Index score										
Baseline	0.72	(0.22)	0.74	(0.17)	0.653	NA		NA	NA	
Posttreatment	0.87	(0.14)	0.80	(0.17)	0.088	0.78	(0.66 to 0.91)	0.014	0.42	(-0.06 to 0.91)
One-year follow-up	0.88	(0.10)	0.81	(0.15)	0.051	0.81	(0.62 to 1.00)	0.052	0.50	(-0.00 to 0.99)
Two-year follow-up	0.91	(0.10)	0.81	(0.18)	0.010	0.82	(0.66 to 0.98)	0.024	0.66	(0.16 to 1.16)
EQ-5D-5L VAS score										
Baseline	56.47	(26.53)	57.03	(23.85)	0.928	NA		NA	NA	
Posttreatment	74.03	(20.01)	68.18	(19.97)	0.236	64.51	(46.94 to 82.08)	0.143	0.29	(-0.19 to 0.77)
One-year follow-up	74.69	(11.14)	62.50	(18.10)	0.002	66.77	(46.89 to 86.66)	0.034	0.81	(0.30 to 1.32)
Two-year follow-up	80.45	(12.59)	63.55	(21.34)	<0.001	69.08	(52.03 to 86.13)	0.003	0.97	(0.45 to 1.49)
LASMI daily living										
Baseline	1.72	(1.07)	1.70	(1.06)	0.935	NA		NA	NA	
Posttreatment	1.01	(0.68)	1.58	(1.05)	0.009	1.49	(1.08 to 1.91)	<0.001	0.66	(0.16 to 1.15)
One-year follow-up	0.98	(0.85)	1.36	(1.03)	0.118	1.35	(0.85 to 1.85)	<0.001	0.39	(-0.10 to 0.88)
Two-year follow-up	0.99	(0.85)	1.31	(0.96)	0.166	1.28	(0.72 to 1.84)	0.002	0.35	(-0.15 to 0.84)
LASMI interpersonal relations										
Baseline	1.63	(0.91)	1.52	(1.01)	0.652	NA		NA	NA	
Posttreatment	0.94	(0.51)	1.47	(0.86)	0.003	1.37	(0.84 to 1.89)	<0.001	0.74	(-0.25 to 1.24)
One-year follow-up	0.97	(0.67)	1.40	(1.06)	0.054	1.27	(0.76 to 1.79)	<0.001	0.49	(-0.01 to 0.98)
Two-year follow-up	1.14	(0.69)	1.41	(1.05)	0.228	1.25	(0.70 to 1.81)	<0.001	0.30	(-0.19 to 0.80)
LASMI work										
Baseline	2.42	(0.59)	1.86	(0.89)	0.053	NA		NA	NA	
Posttreatment	1.37	(1.64)	1.78	(0.96)	0.221	1.88	(0.94 to 2.82)	<0.001	0.30	(-0.18 to 0.78)
One-year follow-up	1.25	(0.80)	1.69	(1.00)	0.055	1.72	(0.82 to 2.63)	<0.001	0.49	(-0.01 to 0.98)
Two-year follow-up	1.36	(0.85)	1.74	(0.95)	0.098	1.66	(0.66 to 2.66)	<0.001	0.42	(-0.08 to 0.91)
LASMI endurance and stability										
Baseline	3.93	(1.31)	3.51	(0.94)	0.127	NA		NA	NA	
Posttreatment	4.10	(1.81)	3.45	(1.48)	0.114	3.75	(2.58 to 4.91)	0.393	-0.39	(-0.87 to 0.09)
One-year follow-up	3.49	(1.09)	3.63	(1.96)	0.732	3.63	(2.51 to 4.74)	0.039	0.09	(-0.40 to 0.57)
Two-year follow-up	3.66	(1.39)	3.35	(1.47)	0.394	3.54	(2.04 to 5.04)	0.067	-0.22	(-0.71 to 0.28)
LASMI self-recognition										
Baseline	2.40	(1.08)	2.22	(1.04)	0.472	NA		NA	NA	
Posttreatment	1.74	(0.99)	1.93	(1.09)	0.467	2.09	(1.39 to 2.79)	0.073	0.18	(-0.30 to 0.66)
One-year follow-up	1.40	(0.76)	1.77	(1.18)	0.139	1.91	(1.06 to 2.77)	0.018	0.37	(-0.12 to 0.86)
Two-year follow-up	1.71	(0.85)	1.79	(1.21)	0.741	1.87	(1.07 to 2.68)	0.033	0.08	(-0.41 to 0.57)
PANSS positive										
Baseline	23.03	(7.63)	22.24	(8.60)	0.688	NA		NA	NA	
Posttreatment	17.53	(6.64)	19.24	(9.91)	0.408	20.39	(16.18 to 24.61)	0.060	0.20	(-0.28 to 0.68)
One-year follow-up	17.12	(6.10)	19.26	(9.24)	0.276	19.53	(14.98 to 24.09)	0.045	0.28	(-0.22 to 0.77)
Two-year follow-up	18.85	(6.37)	20.61	(9.25)	0.375	19.64	(14.88 to 24.41)	0.054	0.22	(-0.27 to 0.71)
PANSS negative										
Baseline	25.62	(5.32)	23.38	(7.91)	0.176	NA		NA	NA	
Posttreatment	14.03	(3.92)	20.30	(8.63)	<0.001	20.61	(16.47 to 24.76)	<0.001	0.94	(0.43 to 1.44)
One-year follow-up	15.30	(5.67)	20.61	(8.74)	0.005	19.57	(14.66 to 24.48)	<0.001	0.73	(0.22 to 1.23)
Two-year follow-up	17.61	(5.78)	22.39	(9.11)	0.014	19.62	(15.12 to 24.12)	<0.001	0.63	(0.13 to 1.13)
PANSS general psychopathology										
Baseline	45.97	(15.04)	46.56	(15.48)	0.874	NA		NA	NA	
Posttreatment	38.56	(14.77)	40.09	(16.76)	0.692	42.48	(35.41 to 49.56)	0.804	0.10	(-0.38 to 0.58)
One-year follow-up	36.94	(13.99)	41.58	(19.10)	0.270	41.11	(32.32 to 49.89)	0.203	0.28	(-0.22 to 0.77)
Two-year follow-up	40.45	(14.38)	41.81	(16.54)	0.728	40.98	(31.94 to 50.02)	0.257	0.09	(-0.40 to 0.58)
PANSS total										
Baseline	94.76	(25.44)	92.62	(30.54)	0.754	NA		NA	NA	
Posttreatment	70.12	(23.96)	77.29	(35.95)	0.336	83.24	(69.57 to 96.92)	0.017	0.24	(-0.24 to 0.71)
One-year follow-up	69.27	(23.47)	81.52	(35.29)	0.105	80.29	(65.55 to 95.04)	<0.001	0.41	(-0.09 to 0.91)
Two-year follow-up	76.91	(24.68)	84.81	(33.58)	0.286	80.42	(65.06 to 95.78)	<0.001	0.27	(-0.22 to 0.76)
mGAF-F										
Baseline	42.06	(9.58)	40.62	(10.70)	0.560	NA		NA	NA	
Posttreatment	50.35	(7.45)	40.58	(16.21)	0.002	43.24	(34.46 to 52.01)	<0.001	0.78	(0.28 to 1.27)
One-year follow-up	54.97	(7.86)	45.94	(14.25)	0.002	45.51	(36.03 to 54.98)	0.004	0.79	(0.28 to 1.30)
Two-year follow-up	54.88	(7.82)	47.61	(15.46)	0.020	46.86	(35.89 to 57.83)	0.009	0.60	(0.10 to 1.10)

Compared to GOT alone, treatment with GOT + IOT resulted in significantly increased improvements from baseline to one year in verbal memory, working memory, verbal fluency, attention, executive function, and composite scores on the BACS. Similarly, treatment with GOT + IOT showed significantly greater improvements from baseline to two years in verbal memory, working memory, verbal fluency, attention, executive function, and composite scores on the BACS. There were significant differences between groups in the changes from baseline to the one-year follow-up in QLS score; VAS score on the EQ-5D-5L; daily living, interpersonal relations, work, endurance and stability, and self-recognition on the LASMI; positive subscale, negative subscale, and total scores on the PANSS; and mGAF-F scores. Significant differences between groups in changes from baseline to the two-year follow-up were observed in QLS scores; index and VAS scores on the EQ-5D-5L; daily living, interpersonal relations, work, and self-recognition on the LASMI; negative subscale and total scores on the PANSS; and mGAF-F.

Risk of rehospitalization

**Figure 2:**
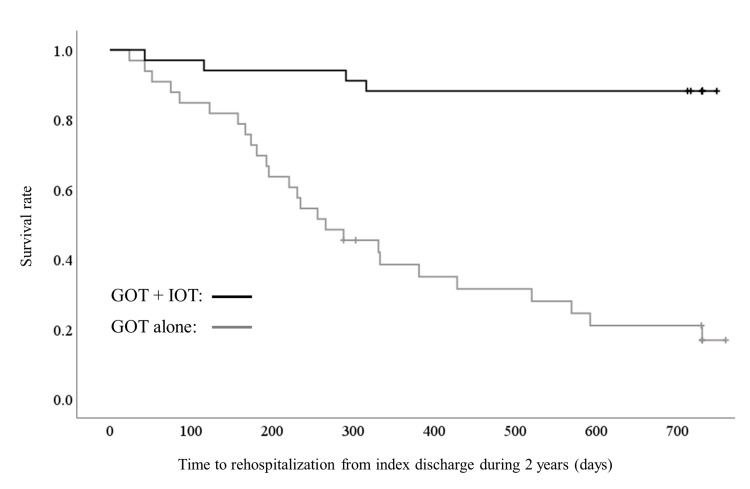
Time to rehospitalization over the two-year follow-up period from discharge (days). Kaplan–Meier survival curve of time to rehospitalization for the GOT + IOT (n = 32) and GOT alone (n = 30) groups (log–rank χ^2^ = 33.19, p < 0.001). GOT: group occupational therapy; IOT: individualized occupational therapy

The mean time to rehospitalization during the two-year follow-up period for all participants was 504.09 (SD = 265.46) days: 666.24 (SD = 180.55) days for the GOT + IOT group and 337.03 (SD = 234.54) days for the GOT-alone group. Over the follow-up period, a total of 27 rehospitalizations occurred (rehospitalization rate = 43.55%): of these, four occurred in the GOT + IOT group (rehospitalization rate = 12.50%) and 23 in the GOT-alone group (rehospitalization rate = 76.67%) (χ^_2_^ = 30.42, p < 0.001). Significantly fewer participants were rehospitalized during the two-year follow-up in the GOT + IOT group than in the GOT-alone group. In addition, the time to rehospitalization was significantly longer for the GOT + IOT group than for GOT-alone (log-rank χ^2^ = 33.19, p < 0.001) (Figure 2).

Participants’ clinical characteristics associated with rehospitalization

The multivariate Cox proportional hazards model showed that type of OT (HR = 0.03, 95% CI = 0.00-0.15, p < 0.001), resident support persons (HR = 0.32, 95% CI = 0.10-0.97, p = 0.045), and medication adherence (HR = 0.33, 95% CI = 0.11-0.95, p = 0.032) were significantly associated with rehospitalization (Table 6).

**Table 6:** Results of multivariate Cox regression analysis on the impact of demographics and clinical factors on rehospitalization of patients with schizophrenia. Multivariate Cox regression was used after controlling simultaneously for potential confounders; adjustment for rehospitalization is based on age, sex, and number of hospital stays. BACS: Brief Assessment of Cognition in Schizophrenia; CI: confidence interval; GOT: group occupational therapy; HR: hazard ratio; IOT: individualized occupational therapy; OT: occupational therapy; SE: standard error

	β	SE	HR	95% CI	Wald	P-value
Type of inpatient OT (GOT + IOT vs. GOT alone)	-3.70	0.91	0.03	0.00–0.15	16.49	<0.001
Physical comorbidities	-1.16	0.70	0.31	0.08–1.23	2.76	0.097
Switching antipsychotics during hospitalization	-0.46	0.49	0.63	0.24–1.66	0.87	0.352
BACS composite score at posttreatment	-0.19	0.19	0.83	0.57–1.20	1.01	0.314
Resident support persons	-1.15	0.57	0.32	0.10–0.97	4.03	0.045
Medication adherence (good vs. poor)	-1.12	0.54	0.33	0.11–0.95	4.23	0.032

IOT components associated with rehospitalization

The type of inpatient OT (GOT + IOT or GOT alone) was significantly associated with rehospitalization; therefore, we tested the impact of IOT components on rehospitalization in the GOT + IOT group. The results of the univariate and multivariate Cox regression analyses revealed no significant components (Table 7).

**Table 7:** Results of univariate and multivariate Cox regression analyses on the impact of IOT components on rehospitalization of patients with schizophrenia. CI: confidence interval; HR: hazard ratio; IOT: individualized occupational therapy; SE: standard error

IOT components	Β	SE	HR	95% CI	Wald	P-value
Univariate analysis						
Number of						
Interview	-0.10	0.10	0.91	0.75–1.09	1.06	0.304
Self-monitoring	-0.04	0.08	0.96	0.82–1.13	0.23	0.629
Individualized visits	0.15	0.09	1.16	0.97–1.39	2.70	0.100
Craft activities	-0.03	0.07	0.97	0.84–1.12	0.22	0.639
Individualized psychoeducation	-0.59	0.59	0.55	0.18–1.74	1.02	0.312
Discharge planning	-0.71	0.66	0.49	0.14–1.80	1.15	0.284
Implementation time of						
Interview	0.00	0.00	1.00	1.00–1.01	0.18	0.674
Self-monitoring	-0.00	0.00	1.00	1.00–1.00	0.14	0.708
Individualized visits	-0.00	0.01	1.00	0.99–1.01	0.53	0.467
Craft activities	0.00	0.00	1.00	1.00–1.00	0.03	0.862
Individualized psychoeducation	-0.02	0.01	0.98	0.96–1.01	2.13	0.144
Discharge planning	-0.01	0.01	0.99	0.98–1.01	0.67	0.414
Multivariate analysis						
Number of						
Interview	-25.98	17.43	0.00	0.00–3.54e+3	2.22	0.136
Self-monitoring	-13.27	8.85	0.00	0.00–58.72	2.25	0.134
Individualized visits	17.58	14.80	4.31e+7	0.00–1.71e+20	1.41	0.235
Craft activities	-21.39	18.37	0.00	0.00–2.23e+4	1.36	0.244
Individualized psychoeducation	-9.57	10.87	0.00	0.00–1.45e+5	0.78	0.379
Discharge planning	-52.98	37.67	0.00	0.00–1.14e+7	1.98	0.160
Implementation time of						
Self-monitoring	-0.06	0.03	0.94	0.82–1.08	0.82	0.366
Individualized visits	0.29	0.89	1.33	0.23–7.62	0.10	0.747
Craft activities	-0.00	0.01	1.00	0.97–1.02	0.08	0.784
Individualized psychoeducation	-0.32	0.95	0.73	0.11–4.66	0.12	0.735
Discharge planning	0.08	0.08	1.09	0.93–1.27	1.13	0.289

Mediating effect of the relationship between inpatient OT type, medication adherence, and rehospitalization

**Figure 3:**
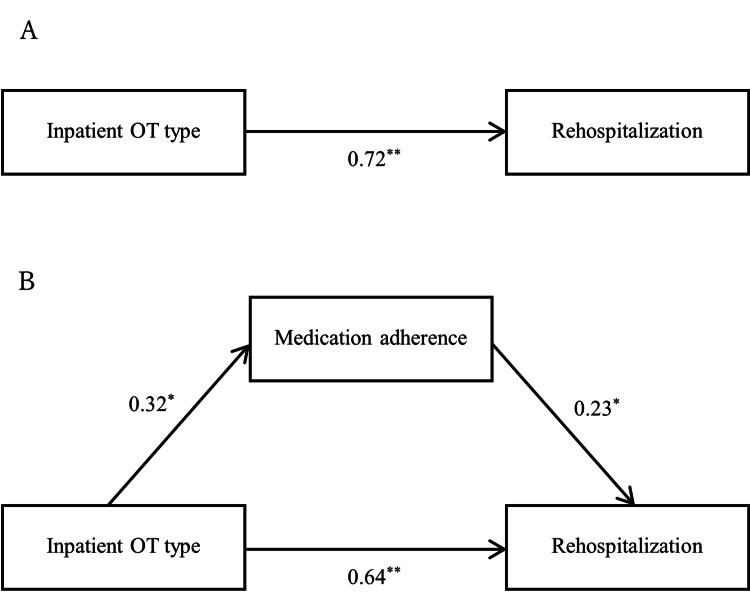
The mediating role of inpatient OT type in the relationship between medication adherence and rehospitalization. A: Model 1; B: Model 2. ^*^: p < 0.05, ^**^: p < 0.01. OT: occupational therapy

A mediation analysis was conducted to examine the relationships among inpatient OT type, medication adherence, and rehospitalization. The estimated value was 0.075 (95% CI = 0.010-0.212). The results showed that the inpatient OT type had a significant effect on rehospitalization (β = 0.72, p < 0.001). The direct effect of inpatient OT type on rehospitalization was significant (β = 0.64, p < 0.001), and the indirect effect of inpatient OT type on rehospitalization via medication adherence was not significant (β = 0.08, p = 0.135). Table 8 and Figure 3 present further details.

**Table 8:** Mediating effects of type of inpatient occupational therapy on medication adherence and rehospitalization. X: type of inpatient occupational therapy; Y: rehospitalization; M: medication adherence; B: unstandardized coefficient; β: standardized coefficient; SE: standard error

Model			B	β	SE	t	P-value
Model 1	X → Y		0.71	0.72	0.10	7.50	<0.001
Model 2	X → M → Y	X → M	0.31	0.32	0.13	2.48	0.016
		M → Y	0.24	0.23	0.10	2.41	0.020
		X → Y	0.64	0.64	0.10	6.63	<0.001

## Discussion

We conducted a two-year prospective cohort study at 14 sites across Japan to evaluate the impact of adding IOT to standard care on rehospitalization. The main finding of this study was that adding IOT to standard care significantly prolonged the time to rehospitalization, and receiving IOT during hospitalization, having resident support persons, and maintaining good medication adherence reduced the risk of rehospitalization of patients with schizophrenia. These results are consistent with those of our prior study [7], which demonstrated that the IOT was effective in preventing rehospitalization.

This study evaluated which IOT components are beneficial to reduce the risk of rehospitalization; however, we could not detect the significant factors related to each IOT component, including implementation number and time, that affected rehospitalization. However, the overall findings of the superiority of IOT provide useful avenues for preventing rehospitalization in patients with schizophrenia.

Furthermore, as a key finding, we identified the relationships among OT type, medication adherence, and rehospitalization and the pathway by which OT type affected rehospitalization. In line with the results of the multivariate Cox regression analysis, we explored the direct and indirect relationships among inpatient OT type, medication adherence, and rehospitalization of patients with schizophrenia. The results of the mediation analyses showed that OT type had a significant and direct effect on rehospitalization as well as mediated the relationship between medication adherence and rehospitalization, confirming the significant role of OT type in rehospitalization. Although OT type had significant direct and indirect effects on rehospitalization, it is noteworthy that the direct effect of OT type on rehospitalization was greater than its indirect effect via medication adherence. This aspect indicates that IOT reduces the risk of rehospitalization not only via improved medication adherence but also via its own effect on the risk of rehospitalization.

Consistent with the results of prior studies [7,21,22], the results of our study showed that medication adherence significantly reduced the rehospitalization risk. Medication adherence is considered an important factor in this regard. Several interventions to improve adherence rates have been developed [23]. However, poor medication adherence is highly prevalent among schizophrenia [22] and is associated with sharp increments in the rates of relapse and rehospitalization [24]. Several factors may have contributed to the results of this study, which showed that good medication adherence significantly reduced the risk of hospitalization. Psychoeducation [25] and discharge planning [26], including IOT, have been shown to be effective in enhancing treatment adherence of patients with schizophrenia. Treatments with individualized psychoeducation and discharge planning may improve treatment adherence and effectively prevent rehospitalization. Findings of this study highlight that the clinical utility of IOT is effective for prompting medication adherence, improving treatment adherence, and thus reducing the risk of rehospitalization.

Compared with GOT alone, GOT + IOT showed significant, sustained improvements in most subscales of social functioning, as measured by LASMI, and in functional level, as measured by mGAF-F, over the one- and two-year follow-up periods. These findings suggest that the IOT intervention has an enduring effect on functional outcomes at one and two years after the inpatient OT intervention. Interventions aimed at enhancing cognition may have an impact on functional outcomes, but intrinsic motivation [27-29] and negative symptoms [30], which were observed to show sustained improvements in this study over the one- and two-year follow-up periods, are critical mediators of the relationship between cognition and functional outcomes. These aspects of schizophrenia constitute distinct and essential therapeutic domains that may be required to generalize cognitive improvements to functional outcomes. Proactive participation in IOT appears to facilitate sustained improvements in cognition, intrinsic motivation, and negative symptoms, providing opportunities for transition to improved functional outcomes. These results, in which the significant improvement in functional outcomes in the GOT + IOT group was maintained, may have strongly contributed to the significantly lower rehospitalization rate in the GOT + IOT group.

Strengths and limitations

This study provides evidence that IOT during hospitalization is effective in preventing rehospitalization among patients with schizophrenia. We recommend the incorporation of IOT intervention to traditional OT based on group treatment for improving rehospitalization rates of patients with schizophrenia. However, we could not verify how components of IOT affected rehospitalization, and further studies are needed to identify the optimal IOT intervention to prevent rehospitalization. The strength of this study is that the IOT, and not the traditional GOT, reduced the rehospitalization rate of patients with schizophrenia. This finding is expected to encourage the revision of the psychiatric OT medical fee system in Japan, resulting in a positive social impact.

This study had some limitations. First, we did not achieve the planned sample size of 96 patients (48 per group) and only included 62 patients (64.58%). The generalizability of the findings may be limited due to the small sample size. Second, the study sample only comprised patients who were discharged within one year of hospitalization. Third, the IOT dosage was adjusted by the occupational therapist in charge according to the patient’s condition and was not standardized. Further studies are needed to explore the optimal dosage, including the duration, frequency, and implementation time of each IOT component, to maximize outcomes. Fourth, we did not use an objective assessment of medication adherence in this study; therefore, the possibility of adherence issues affecting the rehospitalization cannot be excluded.

## Conclusions

We conducted a two-year, prospective cohort study to evaluate whether, compared to GOT alone, the addition of IOT to GOT during hospitalization would lead to the reduction of rehospitalization risk among patients with schizophrenia. Our findings showed that the type of inpatient OT and medication adherence were significantly associated with rehospitalization, and that receiving IOT during hospitalization and good medication adherence could reduce the risk of rehospitalization. This study provides evidence that adding the IOT to standard care is effective in preventing rehospitalization of patients with schizophrenia.
